# 

**Published:** 1874-03-15

**Authors:** 


No. VI. CHICAGO, MARCH, 15, 1874. Vol. XV.

THE

### Medical Examiner.

A

### Semi-Monthly Journal of Medical Sciences.

*TERMS.—Yearly Subscriptions, Three Dollars, in advance. Single Number Fifteen Cents. All Communications should be addressed to Publishers* MEDICAL EXAMINER, 65 *Randolph st., cor. State, Chicago.*

